# Perceptions and experiences of using mobile technology for medication adherence among older adults with coronary heart disease: A qualitative study

**DOI:** 10.1177/2055207620926844

**Published:** 2020-05-20

**Authors:** Linda G Park, Fion Ng, Janet K Shim, Abdelaziz Elnaggar, Ofelia Villero

**Affiliations:** 1 Department of Veterans Affairs Medical Center, USA; 2 University of California, San Francisco School of Nursing, USA; 3 Department of Community Health Systems, University of California, USA; 4 Department of Family Health Care Nursing, University of California, USA; 5 Department of Social and Behavioral Sciences, University of California, USA

**Keywords:** Medication adherence, mobile health, text messaging, mobile applications, focus groups, qualitative research

## Abstract

**Objective:**

Medication non-adherence is linked to adverse clinical outcomes (i.e. rehospitalization, mortality) among patients with coronary heart disease. Given its global adoption and growing popularity among older adults, mobile technology may be an effective strategy to improve medication adherence. The aim of this article is to present the perceptions, attitudes, and beliefs of individuals with coronary heart disease about using text messaging and mobile phone applications for medication adherence.

**Methods:**

We recruited 28 participants (veterans and non-veterans) with a history of coronary heart disease and antiplatelet medication use in Northern California. We formed six focus groups of individuals who participated in three sessions (total 18 sessions). We analyzed our data using grounded theory.

**Results:**

The median age was 69.5 ± 10.8 years for non-veterans (50% male) and 70 ± 8.6 years for veterans (100% male). In the first session, we found that participants perceived text message reminders as a convenient, easy, and flexible tool to establish a routine for taking medications. In the second session, participants were eager to use applications for their greater interactivity, individualized health monitoring, and personalized medication information. The third session, participants shared preferred features (i.e. drug interactions, tracking symptoms) after using two applications at home for 2 weeks.

**Conclusions:**

Older adults are engaged and can be proficient mobile technology users. Text messaging and mobile phone applications are perceived as helpful tools for medication adherence. Future research should include rigorous clinical trials to test the efficacy of mobile health technology to promote medication adherence in populations that require strict medication adherence.

Medication non-adherence is a major public health problem^1^ and a significant barrier in treating illness, with the majority of individuals with chronic disease being non-adherent to some or all of their prescribed medications.^2^,^3^ Among patients with coronary heart disease (CHD), medication non-adherence has been closely linked to adverse clinical outcomes such as rehospitalization and mortality.^4–6^ For example, adherence to antiplatelet (thienopyridine) therapy for the first 12 months after acute coronary syndrome (ACS) or percutaneous coronary intervention (PCI) with a drug-eluting stent is critical in preventing in-stent thrombosis, and potential recurrent myocardial infarction (MI) and death.^7–9^ However, it is estimated that one in seven MI patients stop taking antiplatelet medications by 30 days post-treatment with drug-eluting stents.^9^ Despite the success of multi-modal interventions that involve providers, ensuring strict medication adherence among patients continues to elude healthcare providers.^10^ Therefore, more cost-effective and non-intrusive interventions should be considered to promote medication adherence.

Studies with recent evidence looking at digital interventions for behavior change show promise for those living with chronic disease.^11^ Text-message based interventions have been considered feasible and favorable given their automated administration, long-standing use, and ease of use.^12^ With increasing global adoption, and growing popularity among older adults, mobile technology may be an effective strategy to improve medication adherence.^13^ Mobile health (mHealth) is defined as communicating health data via mobile devices. Two convenient and inexpensive forms of mHealth used to enhance patient communication are text messaging (TM) and mobile applications (apps). TM is popular amongst all age groups and is easy to use. In comparison to TM, mobile apps are more complex and offer additional features such as computational capacity to collect and analyze health-related data in real-time to deliver health and behavioral interventions.^14^,^15^ In addition, mobile apps can offer interactivity, gaming, and feedback, whereas TM cannot. Therefore, to ensure successful application of mHealth initiatives to improve medication adherence, it is imperative that perspectives from end-users (i.e. patients with chronic disease) be integrated into the design and implementation of interventions.^16^

Applying rigorous qualitative methodology is an important step in establishing whether there is potential for mHealth strategies to improve medication adherence among patients with chronic diseases.^17^ Experts and multiple stakeholders agree that a patient-centered approach and an increase in patient knowledge are critically important and necessary for medication adherence.^18^ However, the most effective way to engage patients via mHealth has yet to be determined. For this reason, exploratory methods that can inductively generate findings are needed to contribute to an evidence base for the acceptability and feasibility of mHealth for medication adherence in a population of older adults with coronary heart disease. In this article, we present results from focus group discussions, aimed to explore ideas, perceptions, attitudes and beliefs of patients diagnosed with coronary heart disease regarding the use of TM and mobile phone apps for medication adherence. We hypothesized that our sample, comprising of mostly older adults with coronary heart disease who have varied experience with mHealth, would express interest in using mHealth technology for self-management of their chronic condition.

## Methods

This article represents the qualitative research conducted in a mixed-methods study to inform the development of a 12-month randomized clinical trial (RCT) that will examine mHealth technologies to improve medication adherence. For this qualitative study, we organized three focus groups from a community hospital system and another three from the Veterans Affairs (VA) medical centers in the San Francisco Bay Area and Sacramento. Each focus group met for three sessions, yielding a total of 18 focus group sessions. Two different Institutional Review Boards approved the study, one from the joint university and Veterans Affairs Medical Center review board and the other from the participating community hospital system.

### Inclusion/exclusion criteria

Criteria for inclusion in the focus groups were the following: (a) ≥21 years of age, (b) history of ACS or PCI within one year, and (c) current/former antiplatelet (thienopyridine) prescription. Exclusion criteria included: (a) self-reported cognitive impairment; (b) non-ownership of a smartphone; (c) lack of English proficiency/literacy.

### Recruitment

#### Community hospital participants

Participants with history of ACS or PCI were recruited between February–August 2016 from three cardiac conditioning centers affiliated with the non-profit community hospital system in the San Francisco Bay Area. These centers are located in different cities that are socioeconomically diverse; therefore, recruitment occurred at each site to maximize the socioeconomic variability in the sample. A research associate recruited participants through posted flyers at the venues’ bulletin boards and by distributing handouts about the study. Staff at the cardiac conditioning program also provided the research associate with a list of patients who were eligible. Those who agreed to be recruited were enrolled and provided written consent. We recruited a total of 14 participants from the three cardiac conditioning sites, to form one focus group of 4–5 members per site. The median age was 69.5 ± 10.8 years with seven females and seven males.

#### VA medical center participants

Participants for the VA focus groups were recruited between April 2016–April 2017 from three VA medical centers located in the San Francisco Bay Area and Sacramento. From a registry of 1938 veterans, 277 veterans met inclusion criteria after eliminating duplicates and those without proper addresses. Veterans were sent mailers and were contacted by phone to assess interest. Additional recruitment was done at the San Francisco VA Medical Center through flyer distribution. There were 14 veterans from the three VA medical centers who provided written consent and participated in the focus groups from all recruitment strategies. The VA participants were divided into three focus groups depending on their preference of meeting location. The median age was 70 ± 8.6 years and all participants were male. Table 1 shows a summary of the participants’ demographics.

**Table 1.** Participant demographics.

Patient characteristics (*n* = 28)	Total
Age, mean (SD)	66.9 (9.6)
Female, no. (%)	7 (25)
White race, no. (%)	20 (74)
Married/partner, no. (%)	16 (59)
Employed, no. (%)	8 (31)
College graduate, no. (%)	18 (75)

SD: standard deviation.

#### Focus group sessions

All focus groups were conducted in meeting rooms at the hospital where participants received care or at an affiliated health facility. Sessions were moderated by a qualitative researcher with more than 10 years of experience facilitating focus groups and analyzing qualitative data. The principal investigator and a research associate were present at each session to take notes and assist with logistics. Each session lasted between 1–1.5 h. Each of the three sessions were held 2 weeks apart to avoid burdening participants and to allow 2 full weeks to use two mobile apps between weeks 2 and 3. We held two individual in-person and two phone interviews with participants who missed their focus groups for various reasons such as travel, work, etc. Interviews were conducted by the focus group facilitator using the same topic/discussion guides. All participants were paid US$25 in gift cards or cash vouchers after each focus group session or individual interview (total of US$75 for three sessions).

#### Focus group topics and discussion guides

In accordance with the relative lack of evidence on the acceptability of mHealth interventions to boost medication adherence among an older population, we aimed to keep the focus group discussion guides and the individual interview guides both semi-structured and open-ended. This allowed the research team to guide the discussion to topics that would have translational implications for the mHealth intervention design, as well as participants to surface their own ideas for how that design could best facilitate their medication adherence. Figure 1 shows the topic and timing for each focus group session, and the online Supplementary Material Appendix A includes the individual interview guide.

**Figure:**
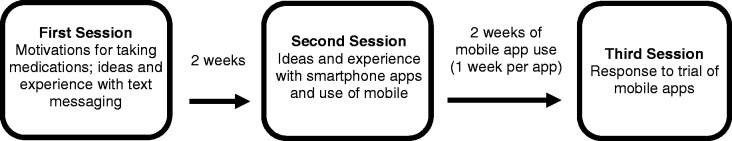
Figure 1. Topics and timing of each focus group session.

The first session was designed to focus on discussions related to participants’ motivations for taking medications and their opinions on the content, frequency, and duration of text messages. This session also explored participants’ ideas for how to overcome text message fatigue and disinterest. The second session focused on smartphone health apps, particularly on participants’ perspective on using them for medication adherence. It also included questions about overcoming barriers on the use of apps for long-term medication adherence. Time was allotted for participants to download two medication reminder apps and learn their basic features at the conclusion of the second focus group. Participants were randomly assigned to use one app for one week and then switch to the other app for the following week. The third session consisted of a review of the use of the two apps.

The two apps that the focus group participants tested were Medisafe and Mango Health. The principal investigator selected these two apps, after reviewing 10 commercially available mobile phone apps. The complete list of mobile apps before prior to narrowing is included in Table 2. The medication adherence apps were graded for their features, ease of use, high ratings from reviewers of health apps, number of downloads, and availability in iOS, Android, and Windows operating systems. Ratings for Medisafe and Mango Health app features were calculated and are presented in Table 3.

**Table 2.** Complete list of mobile applications (apps) reviewed prior to piloting Medisafe and Mango Health.

Mobile app	Description
Health Mapper	Tracks symptoms, medication, and measurements
Symple-Symptom Tracker and Health Diary	Records symptoms over long periods of time
Symptom Tracker	Records and shares symptoms with health care provider, keeps record of prescribed medication and appointments
Health Log	Tracks symptoms and records severity
Health Diary	Tracks vital health parameters (weight, blood pressure, blood sugar, and cholesterol). Adds custom parameters
Flaredown Symptom Tracker for Chronic Illness	Tracks conditions, symptoms, and treatments for chronic illnesses
The Diary-Personalized Health for Improved Care	Tracks blood pressure, BMI, weight, heart rate, medication, etc.
iHealth Log	Tracks medication, dosage, measurements, diary, and reports feature
Mango Health	Tracks medication, dosage, adherence, drug interactions, refill alerts, diary, and provides rewards for adherence
Medisafe	Tracks medication, dosage, drug interactions, vital signs, weight, and blood glucose. Interface with care/provider team, refill alerts

BMI: body mass index.

**Table 3.** Number of participants with respective ratings for favorable application (app) features for both Medisafe and Mango Health.

Rating	Easy to use	Enjoyed getting reminders	Helped me remembermedications
Medisafe			
Strongly disagree	1	3	3
Disagree	0	2	3
Neutral	5	4	2
Agree	8	3	6
Strongly agree	10	10	9
Mango Health			
Strongly disagree	1	4	3
Disagree	3	0	1
Neutral	7	6	4
Agree	6	5	6
Strongly agree	7	4	9

Members of the research team did not have any conflict of interest related to the mobile apps. Medisafe and Mango Health are markedly different in their display windows and offer multiple features to improve medication adherence such as pill reminders, the tracking and monitoring of health measurements such as weight, blood pressure, and cholesterol count, as well as graphs charting medication history and mood.

### Data analysis

All focus group and individual sessions from both institutions were transcribed verbatim by a professional transcriptionist, checked for accuracy, and uploaded to Atlas.ti version 7.0, a qualitative analysis software frequently used in grounded theory.

Analysis was based on a grounded theory approach, which relies on the “emergence of concepts from the data collected.”^19^ Data analysis began with inductive, open coding, which involved the examination of the transcripts to tag actions, events, components of perspectives and experiences, and other data of analytic import for medication adherence itself or the acceptability of TM and mobile apps for medication adherence.^20^ The initial codes were then compared and contrasted for similarities and variations, refined, and in some cases combined or split to build a final set of codes and categories that captured participants’ ideas, perspectives, and experiences with medication adherence using TM and mobile apps.

The same qualitative researcher who facilitated the focus groups also coded and analyzed all of the transcripts, under the supervision of the principal investigator and a co-investigator with expertise in qualitative research. At several points during the coding process, the principal investigator and co-investigator reviewed the emerging codes and data tagged with those codes across the transcripts. These processes helped to ensure that codes were being used consistently and productively to generate findings about older adults’ medication adherence practices as well as acceptability and feasibility of text and smartphone app reminders. The resulting codebook was developed based on their input and iterative review of the data by the main analyst.

## Results

Participants’ perspectives around barriers and facilitators to medication adherence, and the role of TM and mobile apps were explored. Table 4 provides an overview of the most commonly cited themes that pertain to these topics.

**Table 4.** Frequently cited themes from all focus groups.

Familiarity of using TM
App fatigue
Challenges to taking medications: forgetfulness, busy schedule
Desired healthcare provider feedback/interface
Suggested app feature: accountability/family sharing
Suggested app feature: drug interactions

app: application; TM: text messaging.

### Factors affecting medication adherence

Participants from both groups attributed their long-term medication adherence to the establishment of a routine and formation of habit. One female participant from the community hospital group recalled how she started her routine:

At first it wasn’t a routine and I was missing doses when I first got diagnosed … I looked for an app to help me, but I couldn’t find anything. And so then, I had to come up with my own routine where it’s just automatic. It’s gotten to the point where I’ll know, maybe even 5 or 10 minutes before the alarm goes off, that it’s time to take my medicine, and I’m already preparing to take it.

Disruptions in taking medications that were commonly cited were changes in prescriptions or forgetting/missing a refill. Participants attributed their inability to take medications consistently to a lack of routine and/or unanticipated interruptions to their daily routine. An older veteran described one such situation:

There’s something that I’m supposed to take every half-an-hour before I eat, but I never hit that one. It’s the furthest from my mind. I’m a very irregular eater so when I don’t eat or eat whenever, I forget that one. I can’t do it to where I can plug it in at the right times.

Without the use of mobile technology, participants relied on the pillbox system for medication adherence. Participants defined the pillbox system as the use of a pill organizer on a daily, weekly, or monthly basis. Overall, the pillbox system served as tool to track whether they had taken their medications on any given day. However, older participants reported that the pillbox system can be daunting if they had a large quantity of pills. A veteran in his late 70s stated,

I take 11 medications … The pharmacist right here said, when she looked at my pillbox, that it didn’t look like I took my medications. And I said that can’t be right. I look in my pillbox and definitely I didn’t put the Warfarin down … So, it’s complicated, it’s complicated as heck.

A male participant from the community hospital stated: “I’m also a diabetic. But my help is a pillbox. It’s all laid out; I don’t have to think about it. It’s prepared and, so it’s my routine, and I have to keep that routine.”

### TM and medication adherence

Prior to this study, none of the participants had ever used TM as an alert or reminder to take medications, despite that most preferred TM over telephone calls as a primary mode of communication. Similar to general TM, participants noted that the benefits of TM for medication adherence were its ability to allow patients to respond at their convenience, and serve as a physical, time-stamped tool for notes that the patient may not be able to address at a given moment (e.g. while driving). Even patients who have been adherent to medications for years saw the benefit of TM and its potential to address several challenges related to adherence, such as establishing a routine to take medications, combatting forgetfulness and everyday distractions, and populating reminders for appointments and medication refills.

TM was thought to be a potentially helpful tool in not only reminding patients of when, and how much of their medication to take, but also in establishing a routine for participants just beginning a new medication regimen. One participant stated:

Probably my biggest challenge right now is that I went from basically not really taking any medication to now I take about nine, including my vitamins. Just keeping them all straight, and making sure that I’m taking them at the right time, and remembering to get refills and all that stuff are pretty new to me. It’s just been in the last six weeks, so I’m kind of learning a new routine. Text messaging could help with the routine.

While having a pillbox helped with organizing medications, many participants stated that they still have difficulty in taking their pills, given busy schedules, disruptions in daily routines, and “tunnel vision,” that is, starting a task and forgetting about other tasks. Participants voiced that text message reminders, even repeat reminders, could address these barriers. The youngest participant from the community hospital desired a persistent, attention-grabbing TM alert for her busiest hours in the day. She stated:

One time I forgot. It was because I woke up with a phone call … And then I had an appointment that I was supposed to be at, and there was something else that happened. It was like three things that all converged at the same time … So, it’s really the time that I’m the busiest that I need the biggest reminder … Like if it becomes more annoying, or something when it knows that you’re busiest. Because for me, that’s the biggest thing, I have three kids, I have a job, I have stuff that I do for my husband.

Participants also pointed out that TM would also be useful as a reminder system for upcoming clinic appointments and prescription refills, and would be preferred over other reminders such as e-mail or phone call.

### Desired TM features

While a majority of participants see the benefit of text message reminders, they also saw the potential of text message fatigue, which they defined as a feeling of tiredness related to TM that could result in ignored text reminders for taking their medication or alerts being turned off altogether causing them to miss taking their pills.

In addition to providing suggestions to combat message fatigue, participants also desired messages sent from their own medical providers that were personalized to their condition or procedure, instead of an automated text message. A two-way texting interface was also desired, as this allowed for them to engage with the information within the text in a more thoughtful way. One participant shared:

When you respond to a text, you’re much more likely to put that text into your brain as opposed to just a reminder coming up. I promise you after 4 or 5 days of seeing that reminder it’s gonna get ignored because the feeling is, I’m intelligent enough, I know what it is about, I got it, swipe, swipe, swipe. But when it makes you answer, not only does it log on your end so you can keep track, it also makes you read the text message. It just puts it in your brain.

Other desires features include humor to address frustrations related to taking medication. Both focus groups appreciated the idea of a visual photo (e.g. dog) to elicit positive feelings and grab the patient’s attention. One veteran living with chronic illnesses shared, “I’m a dog lover. If I receive a picture of a little puppy, I would pay attention. It would make me smile and make me read the message.” While participants expressed a desire for certain features in their text messages, participants reported negative reactions to messages that would praise them for adhering to their medication regimen, calling them “condescending.” One veteran stated: “It’s kind of, like, okay little boy, and are you gonna do this today? It’s annoying, treating me like a little child.”

### Smartphone apps and medication adherence

A majority of the participants from both groups use smartphone apps extensively and consistently in their daily lives, such as for banking, online shopping, home security, social networking, and more. For those with less frequent use of mobile apps, they still reported having social networking (e.g. Facebook), fitness tracking apps, and games on their phones. However, none of the participants had ever used a medication reminder app.

After consecutive testing of smartphone medication reminder apps for two weeks, one app per week, both groups of participants saw benefits to the use of apps, due to multiple characteristics. First, participants liked the idea of being able to interact with the apps, particularly with the medication reminder. One participant shared:

I liked the fact that it would give you a reminder in 10 minutes if you didn’t take your pills and turn it off. One time I was sitting, reading the paper, and I didn’t feel like getting up to get my phone and turn it off. But then it’d send you a follow-up if you didn’t do anything after two reminders, so I had to take my pills and then go turn it off.

While a majority of participants from both groups experimented with many of the app features, all participants engaged with the pill reminder. Other features that respondents demonstrated interest in were the apps’ medical appointment reminder, advanced dosing schedule, and prescription renewal and refill reminder to ensure enough medication coverage.

Participants also appreciated the apps’ customization and all-inclusive character. Unlike TM, which is limited to issuing simple one or two-way reminders and health messages, smartphone apps offer more comprehensive features like drug information, interactions, and side effects, and could be a repository of a wide range of health measurements (e.g. blood pressure, weight, blood glucose, body temperature, cholesterol levels, and graphs and charts showing a patient’s history of medication adherence). Participants from both groups recognized these features as more compelling and potentially conducive to medication adherence.

Participants shared that an app can provide necessary and crucial information on prescribed medications, such as adverse side effects or changes in formulations that their providers may not have discussed. The ability to track and monitor health indicators on a long-term basis make apps attractive as mHealth platforms. As one community hospital participant stated:

I want to see what my health looks like in the long-term, not just on my day-to-day of just remembering to take my medications. I want to see where my peaks and valleys are … I can see where I need to start tightening up things – visual is a whole lot easier to kind of go, ‘oh gee.’ … But if you have a graph or chart, you know, it’s in your face kind of thing.

While app features such as information on drug interactions and the tracking of health indicators were not directly related to medication adherence, participants expressed strong interest in using these apps to validate and track their health observations and symptoms, and speculated that this heightened incentive to use the apps might in turn boost the use of them for a medication reminder. To this end, many participants viewed the apps as tools for creating and forming a habit of adhering to their medication regimens, as well as help develop better health habits.

### Desired app features

In testing the apps’ multiple capabilities, participants noted that a link to their medical providers would help in medication adherence. One community hospital participant shared:

It would be useful for a physician’s office to keep track of you because the biggest problem is non-compliance. If there’s a way that it would hook up to you, it might be good between an app and the doctor’s office to once in a while make sure that you are taking your medicines, even if you get reminders, it helps to know the doctor will be checking.

The capability of mobile technology to collect a wealth of health information highlighted concerns about possible violations of privacy, such as the hacking of mobile devices and healthcare being a very personal entity, which was frequently shared by several participants. One community hospital participant voiced a strong opinion about safeguarding his heart condition, “This is my health! It’s nobody’s business … My health care is between me and my wife. There needs to be a password code or something, you know, for privacy in those apps.”

## Discussion

Our analysis of 18 focus groups across two different health systems demonstrated that veteran and non-veteran older adults diagnosed with coronary heart disease can benefit from the use of TM and mobile apps for medication adherence. To our knowledge, this is the first study that has examined the perceptions and attitudes of mHealth technology for medication adherence in older adults diagnosed with coronary heart disease. mHealth technology use among younger adults, and in individuals with chronic conditions such as asthma, diabetes mellitus, and depression has been well studied.^21^,^22^ Our findings reveal older adults are engaged and proficient mobile technology users, perceiving TM and mobile phone apps as helpful tools in their medication adherence.

Participants perceived text message reminders as a convenient, easy, and flexible tool to help establish a routine for taking medications, specifically countering forgetfulness and unanticipated distractions or interruptions that occur in daily life, which have been cited as the most common reason for missing doses of medications.^22^ Suggested text message features included non-automated, personalized reminders from the patient’s own provider, as well as integrating humor and images to capture and maintain the patient’s attention. The potential pitfalls of this technology identified by focus group participants mirrored those found in the literature, such as text message fatigue, caused by excessive and frequent text message or mobile app alerts that decrease an individual’s interface with the intervention.^23^ Alert or usage fatigue has been demonstrated across various digital delivery mediums in addition to TM, which include phone calls, digital banners in mobile apps, and push notifications.^24,25^ These alerts have caused physicians to ignore alerts on clinical decision support systems, diminishing the systems’ effectiveness and leading serious adverse consequences for patients.^24^,^25^

After trialing two smartphone apps, participants highlighted the additional advantages of apps that include capabilities of greater interactivity, individualized health monitoring, and personalized medication information (e.g. side effects, drug-drug interactions). Desired app features included a patient and provider interface, allowing medical providers to simultaneously track medication adherence with the patient and, ideally, identify non-adherence promptly.

Given the capacity of apps for storing a wealth of health information, participants noted that a potential downside to smartphone apps was a breach of their privacy. This is a commonly cited barrier in studies examining benefits and barriers to mHealth technology use.^26^ Concerns over the privacy and security of using technology are not uniform among individuals and cut across traditional demographic characteristics (i.e. age, race/ethnicity, urban residence).^26^ This variability may be related to individuals’ technology adoption classification.^26^ Research shows that the consumers generally accept the tradeoffs that come with using mHealth technology; however, they desire to have the final say in what personal information is exchanged and with whom, and in what format.^26^ A trusting relationship with a healthcare provider minimizes the perceived risk whereas communications with businesses (e.g. insurance or Internet companies) via mHealth are perceived as less trustworthy.^26^ A key recommendation for broader dissemination of mHealth for medication adherence is addressing potential concerns of privacy and security upfront by assessing the degree to which consumers perceive a threat and providing a tailored approach for consumers to modify and control privacy settings.^26^

The recent surge of research that has examined mHealth technologies and its effects on medication adherence show promise in improving health outcomes for those living with chronic diseases.^27^ For example, studies demonstrate positive results when looking at mHealth technology interventions, such as improved adherence rates and viral suppression in adult men who have sex with men in anti-retroviral therapy, improved adherence rates and better blood pressure control in adults, as well as in adolescents with asthma who had poor medication adherence at baseline.^28–30^

Findings from a multicenter RCT demonstrated increased medication adherence in MI patients who had a smartphone-based interactive support tool that included an extended version of a drug adherence electronic diary (e-diary) in addition to educational modules, compared to MI patients who only had a simplified version of the drug adherence e-diary and no access to educational modules.^31^ In addition, a systematic review of RCTs aimed to investigate different mHealth tools in improving adherence to cardiovascular disease medications showed all 10 interventions improved medication adherence with mHealth except one heart failure study that found no significant difference in adherence rates between groups using mHealth (smartphone) or telehealth (an ePill box).^32^ These authors concluded that although mHealth holds a capacity for improving medication adherence, mHealth alone, in isolation from other interventions, would not be enough to create large-scale health outcomes.^32^

While studies have demonstrated the promising effects of mHealth technology, there are several important considerations worth mentioning. First, there remains a lack of economic data to support the use of mHealth behavioral interventions; however, it is growing.^33^ In a systematic review of 39 studies spanning 19 countries in upper and upper-middle income countries, many of the studies did not report all recommended economic outcome items, nor did they report items based on a full economic evaluation.^33^ Therefore, it is clear that there is a need to focus on careful reporting and assessment of the economic evaluations of mHealth interventions to support feasibility and sustainability.^33^,^34^ Second, literature has shown gender disparities related to medication adherence, with patients identifying as women being less adherent than men^35^,^36^ for various reasons including perceived personal control and side effects.^37^ This gender disparity has been shown more so with the adult population, and remains consistent across various medication regimens including statin therapy, antiretroviral therapy, and antihypertensive medication.^37–39^ It will be important for healthcare professionals to keep this in mind while assessing internal and external barriers for medication adherence as well as providing education around adherence.^35^,^36^ Third, the effects may not be as significant for adults with chronic disease who are also underserved.^40^ Even though underserved patients can understand the benefits of mHealth, patient-level barriers such as low health literacy, and lack of financial means to purchase the technology lead to inconclusive results on the feasibility of using mHealth technologies as a way to improve medication adherence.^40^ Mixed evidence regarding the benefits of mHealth on medication adherence was cited by authors who conducted a systematic review of 20 articles assessing mobile devices and medication adherence.^41^ The review highlighted improved medication adherence in 65% of the studies, and while that number may seem large and significant, it may be due to the complexities of studying the behavior of non-adherence and its triggers, such as mental illness or lack of support.^41^ Thus, it is worth considering implementing more patient-centered care and promoting more patient autonomy in managing their care, as well as conducting high quality studies that include populations spanning all levels of socioeconomic status and in lower income countries.

The employment of a qualitative approach to this study allowed for a deeper and better understanding of the participants’ engagement with mHealth technologies, which is important for gauging short and long-term engagement with digital behavior-change interventions.^42^ End-users views on interventions’ features such as the look and feel of an app, personalization, and the capacity to tailor content have been shown to increase motivation, and therefore engagement.^43^

This study has several limitations to consider. First, the sample size was relatively small with a total of 28 participants. With respect to the sample population being comprised of older adults with coronary heart disease who were recruited from either a non-profit community hospital system or a VA Medical Center in northern California, it is difficult to determine how reflective these findings are compared to older adults with another chronic disease diagnosis or older adults with a chronic condition living independently out in the community and not within a healthcare setting. There was also a disproportionate number of participants identifying as male compared to female. Of the 28 participants, only seven identified as female. Therefore, generalizability is limited. As stated earlier, literature has shown gender-based differences when it comes to medication adherence. Inclusion of female-identified participants will be needed for future research. Further testing and research are also needed to determine whether the ideas and perceptions generated from this study will be similar to those of a different age group (i.e. middle-aged adults), older adults with chronic conditions in urban and non-urban settings, and those of differing cultural and ethnic backgrounds. In addition, we held two individual in-person and two phone interviews with participants who missed their focus groups for various reasons such as travel, work, etc.; however, we believe the data obtained through the interviews were just as rich or more in depth than those obtained through the focus group setting. Finally, our screening for cognitive impairment was limited to self-report. We will use a screening tool to assess cognitive impairment in our future clinical trial that will examine the use of mobile phone technologies to improve medication adherence.

One potential ethical consideration is that not every older adult with a chronic condition owns a mobile smartphone or is adept at using all the features of smartphone. Therefore, it is important to make technology accessible to older adults who do not own a smartphone. In addition, it is vital to provide adequate training on how to use the features of the smartphones and various health apps that would promote self-management. Introduction to and setup of text message reminders or smartphone apps at the time of diagnosis may enhance medication adherence and set patients up for future success.

## Conclusion

With the constant desire and demand for more diversified and advanced technology, the development of a mobile app and/or TM program to help older adults with chronic conditions with their medication adherence is highly feasible. Older adults with coronary heart disease encounter numerous barriers that impede their abilities to adhere to a medication regimen. Our results suggest that TM and mobile apps could help older adults overcome barriers to medication self-management, like forgetfulness, and everyday distractions.

While mobile technology has traditionally been geared towards younger populations, our study shows older adults can be proficient and “tech savvy” mHealth users. Attention-grabbing text message reminders and interactive mobile apps can increase and deepen patients’ engagement with their medication regimens, potentially reducing the number of rehospitalizations and adverse effects from non-adherence. Future directions for research include rigorous clinical trials to test the efficacy of mHealth technology to promote medication adherence in populations that require strict medication adherence. In addition, economic evaluations (e.g. cost-effectiveness and cost-utility) of mHealth based interventions are needed to assess their scalability and sustainability and help guide policymakers and funders in determining whether evidence supports greater adoption. In addition, it will be vital to include the perspectives and involvement of medical providers and pharmacists to make integration of mHealth technology successful in patient self-management to promote medication adherence. It is also important to address potential concerns of privacy and security while allowing users to modify and control privacy settings.^23^ Finally, incorporating behavior change theory will be critical to design and implement mHealth interventions that promote medication adherence and other behavior change related to secondary prevention.

## Supplemental Material

sj-pdf-1-dhj-10.1177_2055207620926844 - Supplemental material for Perceptions and experiences of using mobile technology for medication adherence among older adults with coronary heart disease: A qualitative study Click here for additional data file. Supplemental material, sj-pdf-1-dhj-10.1177_2055207620926844 for Perceptions and experiences of using mobile technology for medication adherence among older adults with coronary heart disease: A qualitative study by Linda G Park, Fion Ng, Janet K Shim, Abdelaziz Elnaggar and Ofelia Villero in Digital Health
