# Supplementary material for: Perceptions and experiences of using mobile technology for medication adherence among older adults with coronary heart disease: A qualitative study
Source: Digit Health. 2020 May 20;6:2055207620926844. doi: 10.1177/2055207620926844 (PMC7241207; doi:10.1177/2055207620926844)
Supplement: sj-pdf-1-dhj-10.1177_2055207620926844 - Supplemental material for Perceptions and experiences of using mobile technology for medication adherence among older adults with coronary heart disease: A qualitative study [file sj-pdf-1-dhj-10.1177_2055207620926844.pdf]

Appendix: Group Discussion and Individual Interview Guide

SESSION 1: FOCUS GROUP INTERVIEW GUIDE FOR MEDICATION ADHERENCE & TEXT MESSAGING

Medications (10-15 Min)

***** Elicit range of experience, and normalize that there may be differences of opinions

Thank you for the interesting introduction. Now I'd like you to take a moment and think about your medications, specifically the blood thinning medication.

1. What motivates you to take medications?

Probe: knowledge of disease, negative consequences

2. What are the challenges you face in taking medications?

Probes: forgetfulness, cost, don't believe in value

3. And do you have suggestions on how to address the barriers?

4. Do you use any reminders to help you to take medications?

Probes: alarms, spouse, other ways they remember (e.g., always when I wake up, with lunch, etc.)

Mobile Health Questions (30-45 min):

***** Elicit range of experience, and normalize that there may be differences of opinions

1. Do you use text messaging?

2. How do you feel about using text messaging?

3. How often do you text during the day? (a lot, a little, how often?)

4. Can you think of any benefits in using electronic devices to help you with taking your medications?

Probes: useful, useful but don't know how, not useful
Probe for different experiences: "Anyone have some other ideas about benefits?"

5. How do you feel about receiving text messages on your mobile phone to help you with taking your medications?

Probes: useful, useful but don't know how, not useful

6. If you were to receive TM for health, what content in text messages would be most helpful to remind you to take medications?

Probes: Medication-specific information, general health education, cardiovascular health education

7. Who do you want to receive the text messages from?

Probes: clinician, name of coach (female vs. male), buddy

8. What time would be most helpful to received text messages if you were taking medications at different frequencies?

Probes: at the time of medication intake, 5 minutes before, once or twice daily if you are taking a twice daily medication

9. How frequently would you like to receive text messages?

Probes: daily, three times a week

10. Most patients are asked to take blood thinning agents for at least one year. How frequently would you like to receive text messages during the year… more in the beginning and less later on or the same frequency all year round?

11. How can we avoid text messaging fatigue and disinterest?

Probes: change frequency, content, trivia, jokes

12. Do you have suggestions of some text messages you would want to receive?

13. Do you ever get health information through your phone, by text or on an app?

14. Would you have any concerns about getting health info this way?

Probes: privacy/trust issues with technology

15. Would you have any concerns about giving health info back?

Ask only if time allowed (have more than 20 min left)

16. How do you feel about using the internet for health purposes?

Probes: useful, useful but don't know how, not useful

Sample Text Messages (30 min)

Now we want to change gears a little and get your feedback on some ideas on sending text messages to patients to help them take medications.

Now we'd like to share some text messages with you that we have developed and get your reactions and feedback. These are just some examples of the kinds of messages we are thinking about using in the context of a study to see if using mobile phones will be helpful to patients with coronary heart disease. So, we want your honest reactions and suggestions. These would be messages that someone would voluntarily sign up in our research study. You would get a certain # of messages and different kinds of messages over a period of about 12 months. Please note we inserted Plavix as the blood thinning medication in these sample texts. This medication would be substituted for the one you are taking. Also, two-way text refers to the patient responding with a text.

Take a few minutes to read this over. You'll see there are many different kinds of messages. Feel free to make notes on the sheet as you read. If you can please use the code listed on the bottom of each page, that would be great.

Please circle the bullet point next to the texts you like, or put an "X" on the bullet point you don't like, or put a question mark next to the texts you think are a "maybe." Then we can talk about it.

1. What is your initial reaction?

Probe re: number and kind of messages they would want

Let's look at each section in turn.

2. The first section has sample text messages that are longer. What do you think of these? How would you feel about receiving these on your phone?

3. Let's look at the next section. What do you think? How would you feel about receiving these on your phone?

   (go through each section)

4. Do you have any suggestions or thoughts about the kinds of messages that would motivate you to take your medications?

5. Do you have suggestions of some text messages you would want to receive?

SESSION 2: FOCUS GROUP INTERVIEW GUIDE FOR MOBILE APPS

1. Can you describe your overall experience with using mobile apps?

Probes: ease with use, frequency

2. Which apps do you use on your phone and why? (encourage them to look on their phones if necessary)

Probes: weather, health, bank, map

3. Can you remember an app(s) you tried to use in the past and then decided not to use it (them)? Why?

4. What makes you stick with one app and not another?

5. How do you feel about using an app on your mobile phone for health purposes? Specifically to take your medications.

6. Have you used apps on your mobile phone for health purposes?

Probes: tracking

7. What features would you like to see in a mobile app focused on taking care of your health?

8. What features would you like to see in a mobile app focused on helping you to take medications?

9. How long do you think you can faithfully use an app focused on helping you with taking medications?

Provide time to download two apps on their mobile phones. Explain use. Request participants to use one app for one week each (total two weeks).

<u>SESSION 3: FOCUS GROUP INTERVIEW GUIDE FOR EVALUATING MOBILE APPS</u>

Please take a moment to review the Medisafe app before we answer the next set of questions…

APP 1 - Medisafe

1. How often did you use the Medisafe app?
2. How did you feel using the Medisafe app?
3. Which features were the most helpful or enjoyable?
4. Which features were not helpful or enjoyable?
5. What recommendations would you make to improve the Medisafe app?

APP 2 – Mango Health

6. How often did you use the Mango Health app?
7. How did you feel using Mango Health app?
8. Which features were the most helpful or enjoyable?
9. Which features were not helpful or enjoyable?
10. What recommendations would you make to improve the Mango Health app?

General

11. Which app would you use for one year to help you take your blood thinning medication?
12. What are your thoughts about using an app to manage ALL your medications vs. ONLY the blood thinning medication based on your experience over the past 2 weeks?
13. How have your initial thoughts changed about using a mobile app for your health and taking medications after using the apps for two weeks?
14. Would you recommend either app to a friend of family member? Why or why not?
